# Upconverting-photon quenching-mediated perforation influx as an intracellular delivery method using posAuNP@UCNPs nanocomposites for osteoarthritis treatment

**DOI:** 10.1186/s40580-023-00409-y

**Published:** 2024-01-03

**Authors:** Hye Jin Kim, Hui Bang Cho, Hye-Ryoung Kim, Sujeong Lee, Ji-in Park, Keun-Hong Park

**Affiliations:** Laboratory of Nano-Regenerative Medicine, Department of Biomedical Science, College of Life Science, CHA University, CHA Biocomplex, 335 Pangyo-ro, Sampyeong-Dong, Bundang-gu, Seongnam-si, 13488 Republic of Korea

**Keywords:** Upconversion nanoparticles, Au nanoparticles, Intracellular delivery, Photoporation, NIR, Osteoarthritis, Baricitinib

## Abstract

Photoporation techniques based on plasmonic nanoparticles such as gold nanoparticles have been extensively studied for the intracellular delivery of substances via cell membrane disruption. However, the clinical application of AuNP is challenging due to its absorption in the 500 nm region of the light spectrum. To overcome this challenge, upconversion nanoparticles were employed to stimulate AuNP at NIR wavelengths. posAuNP@UCNPs nanocomposites were produced by coating 30 nm UCNPs on 80 nm AuNPs using DOPA-PEI, which were then irradiated with 980 nm NIR light to facilitate their intracellular delivery. TEM and DLS confirmed that posAuNP and UCNP combine to form nanocomposites. Additionally, multiphysics simulation was used to analyze the distribution of the posAuNP electric field based on morphological differences that change as the UCNP ratio increases. Next, effective LED irradiation conditions were established by applying upconverting-photon quenching-mediated perforation influx to C28/I2 cells as suspensions or spheroids. posAuNP@UCNP nanocomposites were confirmed to be effective for the delivery of baricitinib as a treatment for osteoarthritis in a three-dimensional osteoarthritis model. Finally, chondrocyte differentiation was induced through intracellular delivery of baricitinib using posAuNP@UCNPs. The findings suggest that posAuNP@UCNPs have great potential as a tool for non-invasive drug delivery via UCPPin.

**Graphical Abstract:**

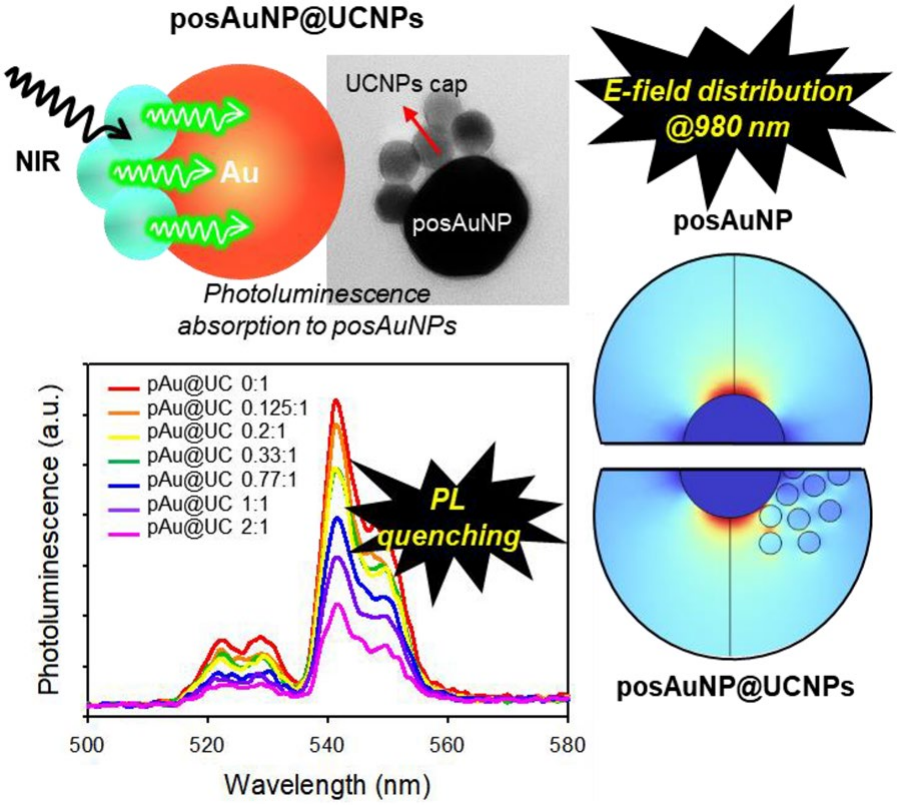

**Supplementary Information:**

The online version contains supplementary material available at 10.1186/s40580-023-00409-y.

## Introduction

Intracellular delivery (ICD) is a technology that delivers therapeutic substances into target cells. This technology is closely related to the fields of cell therapy and regenerative medicine [1, 2]. To enter cells, small molecules such as peptides and nucleic acids must pass through the plasma membrane via passive or active transport [3]. Methods for increasing the efficiency of intracellular delivery of substances have been developed in recent decades [4–6]. Various techniques have been developed, such as carrier-mediated and membrane disruption-mediated methods. Membrane disruption-mediated methods designed to create mechanical fields in plasma membranes are used to form temporary pores [7–9]. These include electroporation [10, 11], sonoporation [12], and photoporation [13–15]. Many studies have reported an increase in the efficiency of membrane disruption using plasmonic nanoparticles [16].

Plasmonic nanoparticles, such as polydopamine nano-sensitizers (IONPs) and gold nanoparticles (AuNPs) irradiated with a 561 nm laser light, have been used to effectively deliver plasmid DNA (pDNA) to HeLa and Jurkat cells [17–20]. Other examples include the delivery of FITC-Dextran and siRNA via AuNPs to HeLa cells and H1299 lung carcinoma cells [21] and the delivery of siRNA to ARPE-19 retinal cells using gold nanostars [22]. Similarly, calcein, an impermeable dye, was delivered to human prostate carcinoma cells and rat cardiomyoblast cells using carbon nanoparticles [23].

AuNPs have been used in several photoporation studies [24] due to their ability to promote the plasmon reaction and create a local electrical field, as well as their high biocompatibility and absorption efficiency [25–28]. During photoporation, the vibration of AuNPs generates heat in a localized area. The high thermal conductivity of AuNPs allows for an instantaneous temperature rise, and a vapor nanobubble is formed. This process aides in the formation of transient pores in the plasma membrane [29–31]. A widely used form of AuNPs called nanospheres are less than 100 nm in size and absorb light in the 500 nm wavelength range. However, since light in the 500 nm range has a relatively short wavelength, it does not effectively penetrate the tissue, limiting its clinical application. Near-infrared light (NIR, 750–2000 nm) has a longer wavelength band and thus, can penetrate tissue and is most suitable for clinical application [32–34].

Upconversion nanoparticles (UCNPs) absorb light in the NIR wavelength band and emit visible light [35, 36]. These nanoparticles are formed by doping sodium ytterbium tetrafluoride (NaYF_4_) nanocrystals with the lanthanide elements ytterbium (Yb^3+^), erbium (Er^3+^), and thulium (Tm^3+^). The combination and arrangement of doping elements and the doping efficiency determine the wavelength and quantum field of light emitted by the NaYF_4_ nanocrystals [37–39]. Unlike photoluminescence, which has a general Stokes reaction (λ_ex_ < λ_em_), the anti-Stokes (λ_ex_ > λ_em_) reaction results in upconverting photons [36, 40–42]. UCNPs are utilized in bioimaging, biosensing, and nanomedicine due to their high light stability [43–47].

**Scheme. 1:**
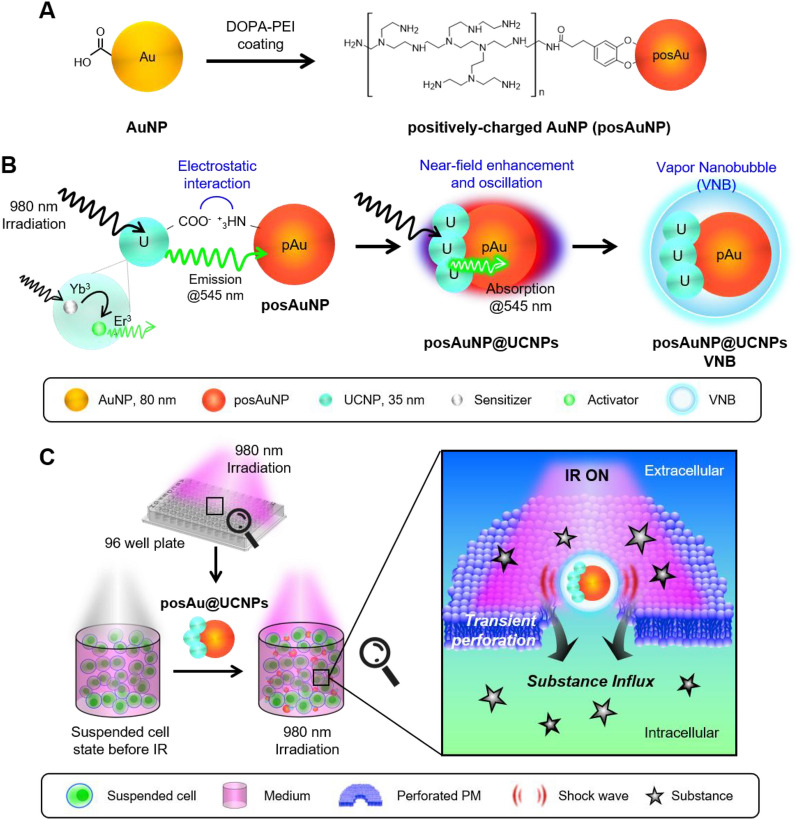
Schematic diagram of the formation process and action of posAuNP@UCNPs. **A** posAuNP was prepared using DOPA-PEI on bare AuNP. **B** posAuNP was then combined with carboxyl-UCNP to form posAuNP@UCNPs. Panel B shows the absorbance of UCNP at a wavelength of 980 nm and the light emitted at a wavelength of 545 nm. Subsequently, a process in which posAuNP absorbs light of 545 nm occurs. **C** The upconverting-photon quenching-mediated perforation influx (UCPPin) process that occurs in cells

This study aimed to produce nanocomposites (NCs) composed of AuNPs and UCNPs and to develop a new photoporation-based ICD method. We synthesized posAuNP caped with UCNPs (posAuNP@UCNPs) nanocomposites with different UCNP ratios and compared and analyzed the morphological and physical properties of these posAuNP@UCNPs. Additionally, we verified that the upconverting photons are quenched by AuNPs and evaluated the temporary perforation of the plasma membrane. This new photoporation technique, called upconverting-photon quenching-mediated perforation influx (UCPPin), was used to deliver baricitinib as a treatment for osteoarthritis in an osteoarthritis (OA) 3D model to verify its applicability in the field of regenerative medicine (Scheme 1).

## Experimental section

### Materials

UCNP-PEG-COOH (QUN91W11) was purchased from ACS Material (California, USA). AuNP (GC80) was purchased from ACS Material BBI solution (UK). Polyethyleneimine (25 k), propidium iodide (P4170), and calcein (C0875) were purchased from Sigma Aldrich (St. Louis, USA). DMEM F12, FBS, and DPBS were purchased from HyClone (Utah, USA). Hoechst 33342 (H3570) and Calcein-AM (65-0853-78) were purchased from Invitrogen (California, USA). STAT1 and STAT3 antibodies were purchased from Santa Cruz Biotechnology (Dallas,Texas, USA). P-STAT1 and P-STAT3 antibodies were purchased from Cell Signaling Technology (Danvers, Massachusetts, USA).

### Preparation and characterization of posAuNP@UCNPs

10 μg of AuNP solution to transferred to an E-tube, and after addition of 7 μg DOPA-PEI, the solution was vortexed at full speed to prepare posAuNPs. If fluorescence observation is required, the posAuNPs were prepared using RITC-DOPA-PEI. The posAuNP 10ug was fixed to 1, UCNP was added at different ratios (0, 1, 2, 5, 10 μg) and vortexed at maximum speed to produce posAuNP@UCNPs.

Each nanocomposite solution (10 μL) was dropped onto a TEM grid formvar surface, allowed to adhere for 10 min. The remaining solution was thoroughly removed using 3 M paper and the TEM grids were dried. The remaining NP solution was measured using DLS.

### IR Effect on posAuNP@UCNPs

posAuNP and posAuNP@UCNPs (1:5) were prepared in a glass vial, and two nanocomposites were simultaneously investigated with an LED emitting light at 980 nm. Next, the temperature was measured at each time using an IR thermometer (TG267, FLIR, USA).

TEM photography was performed to determine the shapes of the NPs with and without LED irradiation. Samples were preparation for TEM observations in the same manner as mentioned above.

The electric field (E-field) and resistive heating losses (RHL) formed by the NPs were calculated and were presented using the Wave Optics module of COMSOL Multiphysics software V. 6.1. NP shape according to the UCNP ratio was examined in TEM images to create a three-dimensional geometrical structure. In addition, a part corresponding to a quarter of the sphere was cut off and perfectly matched layer (PML) was set [48–50]. E-field was implemented by entering the wave equation, assuming that plane waves of 980 nm (UCNP λ_ex_) and 545 nm (UCNP λ_em_) are applied in the X axis direction, respectively, while placed as XZ planes. Parameter information: AuNP radius, 40 nm; UCNP radius 10 nm; wavelength (lda), 980 nm; thickness of air, lda/6; thickness of PML, lda/6. RHL was also calculated under these conditions.

### Localization of posAuNP@UCNPs

C28/I2 cells were harvested with trypsin/EDTA, re-suspended, and then transferred to an E-tube so that each group had 1.0 × 10^5^ cells mL^−1^. Cells were treated with each nanocomposite, left at 4 ℃ for 20 min, and then fixed with 4% PFA before Cell TEM analysis at Eulji University (Seongnam-si, Korea).

C28/I2 cells were attached to the coverslip for one day and then treated with posAuNP@UCNP (1:5) for 30 min. Then, the remaining NPs were removed and fixed with 4% PFA for 1 h and 2.5% glutaraldehyde for 2 h. After washing three times with PBS, dehydration was performed in 50%, 70%, 80%, 90%, 95%, and 100% ethanol solutions for 10 min each. After the HMDS solution was infiltrated and completely dried, it was observed using Tabletop-SEM (SNE-4500 M plus, SEC, Korea).

### Live imaging for transient perforation and re-sealing

1.0 × 10^5^ C28/I2 cells were seeded into a 30 mm dish for CLSM (Olympus, Japan). The next day, the PM was dyed with PlasMem Bright Green (Dojindo) for 20 min. After processing with posAuNP@UCNPs 1:5, a long-distance LED irradiation system was installed to conduct time-lapse imaging with LED IR. Using a live incubation system, real-time images were taken every 6.5 s for up to 20 min while the CLSM chamber was maintained at 37 ℃ and CO_2_ 5%.

To confirm PM re-sealing, the intracellular calcium level was measured with Fluo-4. Using a live incubation system, real-time images were taken every 9 s for up to 15 min while the CLSM chamber was maintained at 37 ℃ and CO_2_ 5%.

### UCPPin efficiency

UCPPin efficiency was analyzed in cell suspensions and spheroids. C28/I2 cells were harvested with trypsin/EDTA, re-suspended, and transferred to E-tubes so that each group had 1.0 × 10^5^ cells mL^−1^. In addition, 5 μg Calcein, 2 μg PI, and 2 μg of each nanocomposite were added to the cells, incubated at 4 °C for 10 min, and then LED IR was initiated. After IR, the remaining dyes and nanocomposites were removed by washing with DPBS and the cells were analyzed with FACS.

For spheroid cell analysis, harvested C28/I2 cells were separated into E-tubes at a concentration of 1.0 × 10^5^ cells mL^−1^. The cells were treated with each nanocomposite, incubated at 4 °C for 10 min, and then washed with DPBS. The cells treated with each nanocomposite were re-suspended in DMEM F12 medium and divided into 10 wells in a u-bottom 96 well plate, and cultured for one day. On the next day, spheroid formation was examined, and Calcein and PI were added before LED irradiation. The cells were then washed with DPBS before CLSM.

### In vitro* OA 3D modeling and analysis*

Nanocomposites, OA Indus (LPS 1 μg mL^−1^ and IL-6 20 ng mL^−1^) and 0.5 μM of BRN were added C28/I2 cells suspended in DMEM F12 serum and anti-biotics free medium. The cells were incubated at 4 °C for 10 min, and irradiated with LED for 20 min. After washing with DPBS, the cells were lysed with RIPA buffer (with proteinase/phosphatase inhibitors) and STAT induction was confirmed by Western blotting.

In other analyses, cells were washed after IR, re-suspended in DMEM F12 medium, divided into six wells in a u-bottom 96 well plate, and cultured for up to 2 weeks. On Day 1 of 3D culture, the IL-6 cytokine level of cells was measured using an IL-6 ELSA kit (430501, BioLegend, USA) according to the manufacturer’s protocol, after diluting the cells three times.

In the first week of 3D culture, western blotting was performed using cells lysed in RIPA buffer. In the second week, spheroids were moved to a 35 mm dish for CLSM, attached to the bottom of the dish, and IF was performed the next day.

### Statistical analysis

Statistical analysis was performed using the student’s t-test in SigmaPlot software 10.0. *P < 0.05, **P < 0.01, and ***P < 0.001 were considered to indicate statistical significance.

## Results and discussions

**Fig. 1:**
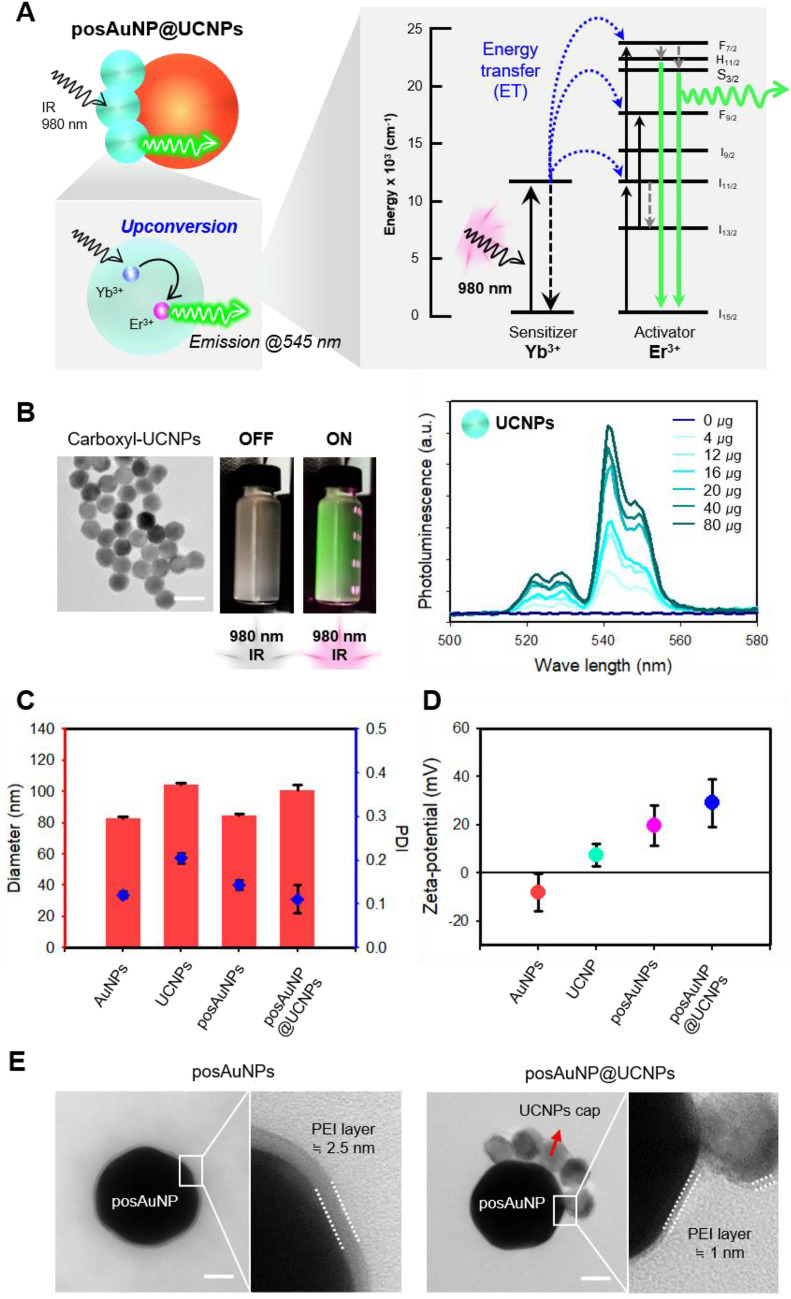
Characterization of UCNP and posAuNP@UCNPs for photoporation. **A** Schematic diagram of the upconversion mechanism of UCNPs. **B** Photoluminescence intensity of UCNP at different concentrations. Scale bar, 40 nm. **C** Size distribution of UCNP, posAuNP, and posAuNP@UCNPs. **D** Zeta potential of UCNP, posAuNP, and posAuNP@UCNPs. **E** TEM images of posAuNP and posAuNP@UCNPs. Scale bar, 20 nm

The UCNP (NaYYbErF4@PEG-COOH) used in this study is a nanoparticle composed of ytterbium (Yb^3+^) as a sensitizer and erbium (Er^3+^) as an activator. The Yb^3+^ ions in the UCNP absorb light at 980 nm, which triggers an anti-Stokes reaction and results in the emission of light at 545 nm (green light) (Fig. 1A). This phenomenon is known as upconversion, and it allows the UCNP to convert low-energy near-infrared light (NIR) to high-energy visible light, making it a useful tool for various applications such as imaging and therapy. This phenomenon is called an upconversion process (anti-Stokes emission reaction) and there are three main types: excited state absorption (ESA), energy transfer upconversion (ETU), and photon avalanche (PA). Among these, the UCNPs used in this study follow the ETU process, which is achieved through energy transfer between two adjacent photons (sensitizer and activator) [51–53]. Energy transfer occurs from excitation of the sensitizer to higher excitation of the activator and continues at higher excitations until the final fluorescence state. Yb3 + , used as a sensitizer, generally has a large absorption cross-section in the NIR region, so it can transfer energy to Er3 + , an activator.

Using TEM, we observed that the UCNPs had a uniform size of approximately 30 nm and emitted light at 545 nm when irradiated at 980 nm. Photoluminescence intensity increased proportionally to the amount of UCNPs (Fig. 1B).

In this study, we measured the size and zeta potential of the AuNPs, UCNP, posAuNP (positively-charged AuNPs), and posAuNP@UCNPs (Fig. 1C, D). Determining the zeta potential of a particle is an important factor in photoporation efficiency, as the process is more effective with cationic particles than anionic particles. This is because cationic nanoparticles interact better with negatively charged cell membranes than anionic nanoparticles [54, 55]. Specifically, Au nanoparticles must be located in the cell membrane to induce stable perforation. This is because the light-stimulated plasmon resonance reaction and VNB formation of Au NPs must occur continuously on the cell membrane surface. Therefore, we utilized 3,4-dihydroxy-l-phenylalanine-conjugated polyethyleneimine (DOPA-PEI) (Additional file 1: Fig. S1) to modify the surface of the AuNPs and make them positively charged (posAuNPs). The UCNP particles have a tendency to aggregate in aqueous solutions despite their small size; however, when coated with UCNP, the size of the AuNPs increases to around 100 nm, and the zeta potential becomes positive (approximately + 30 mV).

TEM observations of the posAuNP and posAuNP@UCNPs revealed that the positive surface charge of the AuNPs was due to the DOPA-PEI layer (Additional file 1: Fig. S2). We confirmed this result using FTIR spectroscopy, which showed the characteristic NH stretching of PEI. When the UCNP was coated on the posAuNP, it tended to stick to only one side of the AuNP (Fig. 1E).

**Fig. 2:**
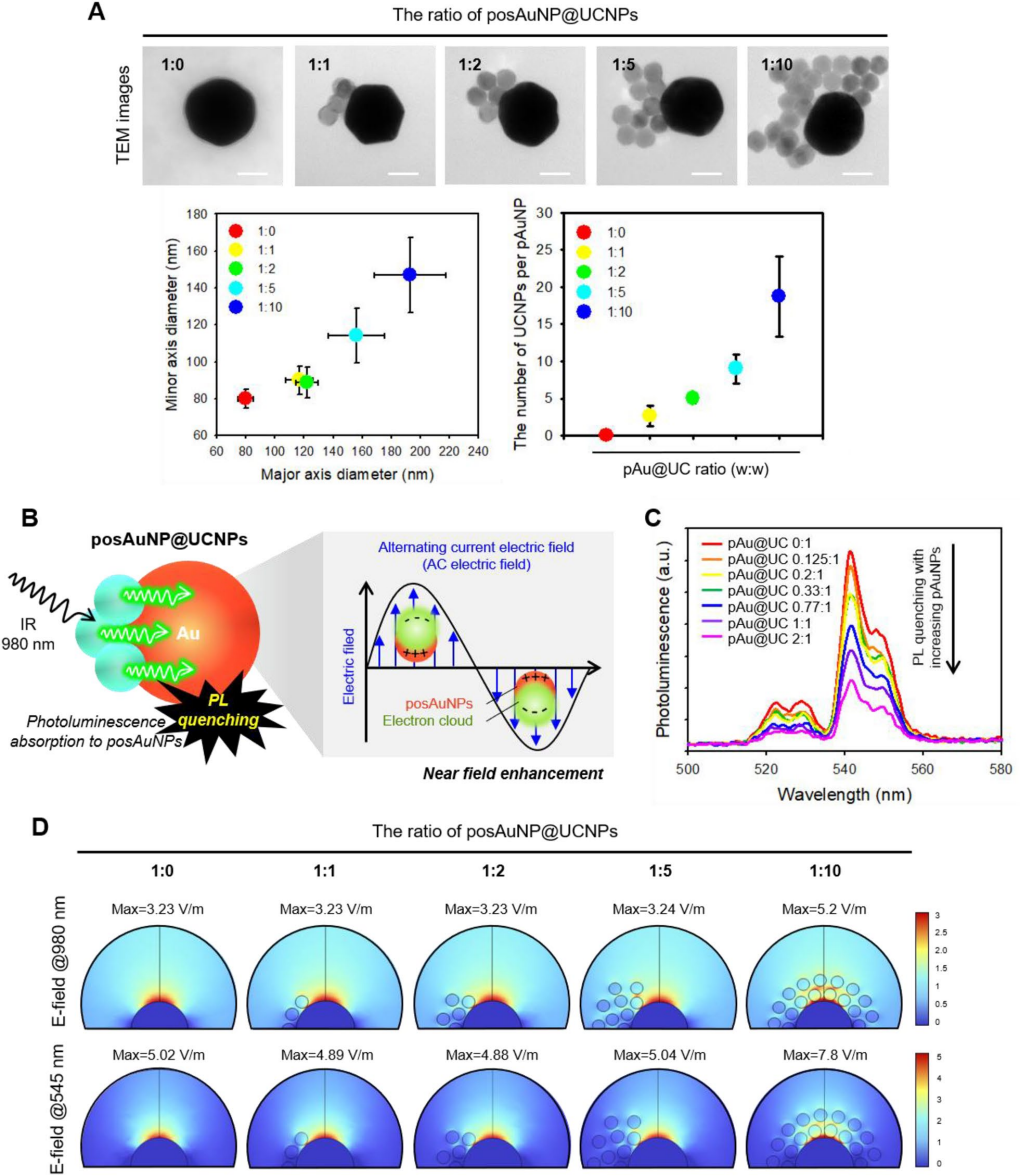
Morphological and physical properties of posAuNP@UCNPs with different UCNP ratios. **A** TEM images showing the morphological properties of posAuNPs at different UCNP ratios. The ratio of UCNP to posAuNP was varied from 0 to 10 to analyze the effect of the ratio on the morphology and properties of the particles. Scale bar, 40 nm. **B** Schematic diagram illustrating the excitation and upconversion emission of UCNPs and the plasmonic absorption of AuNPs. This illustrates how the 545 nm upconverting photons emitted by UCNP can be absorbed by AuNP, leading to photoluminescence quenching. **C** Photoluminescence intensity of posAuNP@UCNPs hybrids as a function of posAuNP ratio. **D** FEM simulations showing the E-field distribution of posAuNP@UCNPs hybrids as a function of UCNP ratio at wavelengths of 980 nm and 545 nm

To determine the optimal ratio of UCNPs to posAuNPs, we fixed the mass ratio of posAuNPs at 1 and varied the UCNP ratio from 0 to 10. We observed that the morphology of the posAuNPs changed according to the UCNP ratio using TEM (Fig. 2A). When the UCNP ratio was increased to 1, 2, and 5, only 1/4 of the posAuNP cross-section was coated with UCNPs on the surface. In the 1:10 group, a form where more than half of the cross-part of NP was coated with UCNPs was observed, and a form that was well bonded to NP even if the UCNP ratio was increased was confirmed. We measured the diameters corresponding to the major and minor axes, which are not evenly bonded to all surfaces (Fig. 2A, lower panel). As the UCNP ratio increased, the number of UCNPs bound to posAuNPs also increased.

Next, we examined the physical properties of the posAuNPs coated with UCNP. UCNPs are excited by 980 nm light and emit an upconverting-photon at 545 nm, while AuNPs are plasmonic nanoparticles that absorb light and form a local E-field (Fig. 2B). Since the wavelength band of 545 nm is similar to the maximum absorbance of 80 nm-sized AuNPs at 550 nm (Additional file 1: Fig. S3), we hypothesized that the 545 nm upconverting photons emitted from UCNPs would be absorbed by AuNPs, resulting in photon quenching. To verify this hypothesis, we measured photoluminescence intensity by fixing the UCNP ratio at 1 and increasing the posAuNP ratio (Fig. 2C). We found that photoluminescence intensity gradually weakened as the posAuNP ratio increased, confirming that photon quenching occurred as a result of re-absorption of the upconverting photon emitted by UCNP by posAuNPs.

We conducted theoretical simulations using the finite element method (FEM)-based Wave Optics module of COMSOL Multiphysics software to investigate the effect of UCNP coating on the electric field (E-field) formed by posAuNPs. We created a three-dimensional geometry at each UCNP ratio, based on the TEM image shown in Fig. 2A. Then, we applied each wavelength range of 980 nm and 545 nm to the X axis in the X–Z plane, which is a perfectly matched layer (PML), and the resulting E-field is shown in Fig. 2D. For 1:0, 1:1, 1:2, and 1:5 ratios, the maximum E-field value at 980 nm was around 3.23 V/m, indicating that UCNPs at these ratios had little effect on the E-field. However, at a 1:10 ratio, which is sufficient to cover half of the cross-section of a posAuNP, we observed a slight increase in the local area near the UCNP. Additionally, at 545 nm, a similar pattern was observed, but the overall increase in the local area was 50% higher than that that observed at 980 nm. These findings suggest that UCNPs have little effect local E-field formation by posAuNPs and that posAuNP@UCNPs maintain the characteristics of plasmonic NPs without affecting their function in the upconverting-photon quenching-mediated perforation process.

**Fig. 3:**
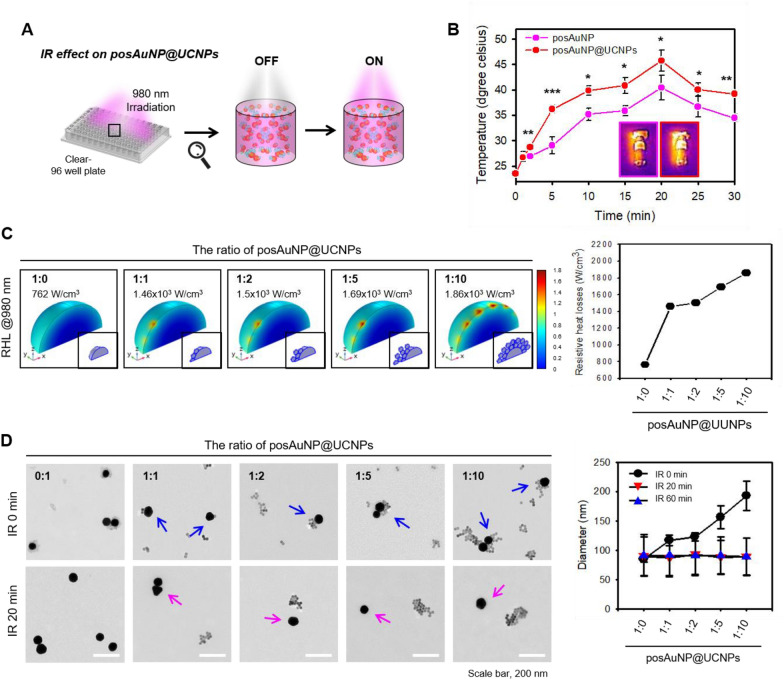
Confirmation of resistive heating losses (RHL) and morphological changes in posAuNP@UCNPs under IR irradiation. **A** Schematic illustration of the experimental setup for IR irradiation of posAuNP@UCNPs and UCNP-coated posAuNP@UCNPs. **B** Heating generation of posAuNP@UCNPs (1:5) compared to posAuNP under conditions irradiated up to 30 min. **C** Graph showing the RHL generated by posAuNP@UCNPs under IR irradiation. The RHL was measured using COMSOL. **D** TEM images of posAuNP@UCNPs and UCNP-coated posAuNP@UCNPs under IR irradiation for different durations. Blue arrows indicate posAuNPs@UCNPs and pink arrows indicate detached posAuNPs from the UCNP cap

Experiments were conducted to evaluate the effect of irradiating posAuNP@UCNPs with 980 nm light (Fig. 3A). The temperature changes that occurred when posAuNP@UCNPs (1:5) were irradiated with 980 nm light were measured for 30 min and were compared with those of irradiated posAuNPs (Fig. 3B). From 2 min after irradiation, posAuNP@UCNPs showed greater heat generation than posAuNPs, and the temperature increase was about 20–35%.

When posAuNP@UCNPs are irradiated with electromagnetic waves, the temperature increases due to the resistance generated, resulting in a physical phenomenon known as resistive heating losses (RHL). It can be interpreted that as the RHL value increases, more conversion to heat energy occurs. In other words, it can be determined that a lot of plasmon resonance reactions of the nanocomposite occurred. Therefore, it was used as an indicator that the photoporation effect would be high. To confirm this, a simulation was performed using COMSOL Multiphysics software (Fig. 3C) using the same structures and conditions as those used in the previous E-field analyses. The simulation showed that a RHL of about 762 W/cm^2^ was generated evenly on the surface of posAuNP@UCNPs in the 1:0 ratio group, while a RHL of 1460 to 1860 W/cm^2^ was generated in the local areas of all the other groups. These values are about 1.9- to 2.4-fold higher, respectively, than that of the 1:0 ratio group, indicating that a large resistance is generated in the local area where the UCNP is bound, leading to heat generation. The occurrence of exotherm is a critical factor for AuNP photoporation because it leads to the formation of vapor nanobubbles (VNBs), which burst and perforate the cell membrane. Therefore, this result confirms that UCNP coating of AuNPs increases the formation of VNBs by increasing the RHL of the AuNPs, ultimately leading to higher UCPPin efficiency.

**Fig. 4:**
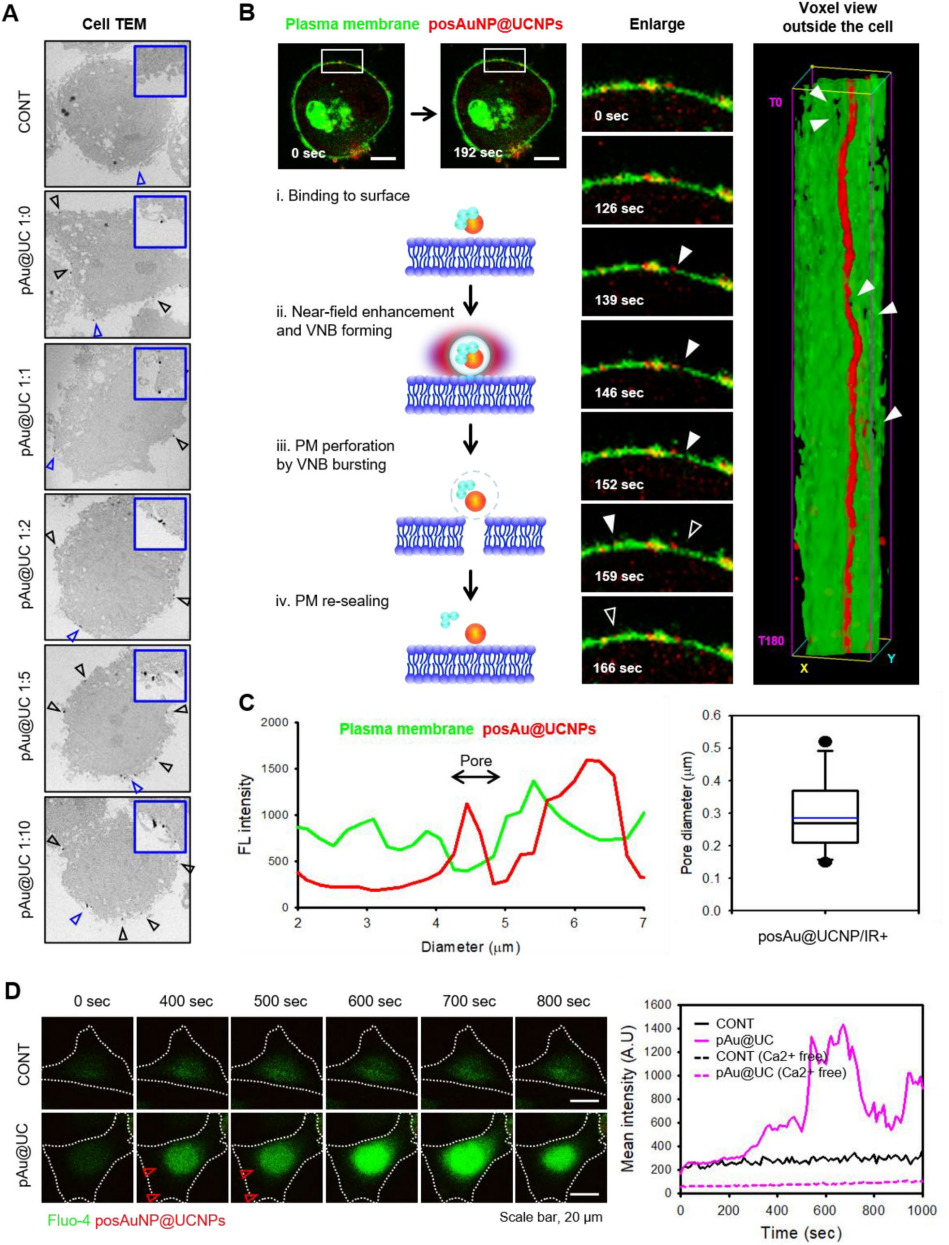
Observation of posAuNP@UCNPs localization and confirmation of transient perforation and re-sealing of the plasma membrane in C28/I2 cells under IR irradiation. **A** Observation of posAuNP@UCNPs localization after 10 min on the NP-treated C28/I2 cells (suspension status). **B** Time-lapse, voxel images, and diagram of transient perforation and re-sealing under IR under conditions after irradiation in C28/I2 cells for up to 20 min (adherent status). Images were taken every 10 s from T0 (0 min) to T180 (20 min). White filled arrows indicate the pore formation area on the plasma membrane and white hollow arrows indicates the re-sealing area after pore formation. This set used posAuNP@UCNPs 1:5. Scale bar, 5 μm. **C** The graph of line profiling from B 159 s and pore diameter. The blue line indicates the average value. **D** Changes in intracellular calcium levels over time after treatment of C28/I2 cells with posAuNP@UCNPs 1:5. Red arrows indicate posAuNP@UCNPs 1:5

To investigate how the above results relate to morphological properties, TEM was used to observe posAuNP@UCNPs before and after IR irradiation (Fig. 3D). In the IR 0 min group, posAuNP and UCNPs were well bonded (blue arrows). However, in the IR 20 min group, the UCNPs had dissociated from the posAuNP@UCNPs and were present in a free state (pink arrows). The change in size was measured by DLS (Fig. 3D, right panel), which showed that the nanoparticles were separate from each other and remained dispersed in a free form until IR 60 min. Additionally, the power (mW/cm2) according to the distance from the light source and the fluence (J/cm2) representing the intensity of energy applied to the area according to the irradiation time were measured and are shown in Additional file 1: Fig. S4A. The size distribution according to irradiation time was estimated by performing an absorbance scan, as described for the Fig. 4D (Additional file 1: Fig. S4B).

The effects of PosAuNP@UCNPs on human-derived chondrocyte line C28/I2 (an adherent cell) cell membranes were observed after electromagnetic irradiation at 980 nm. After treatment with each nanocomposite in re-suspended culture medium, the cells were fixed for 20 min and TEM images were taken. The TEM images showed that all treated NPs were attached to the cell membrane surface (Fig. 4A). The same result was confirmed by SEM when the cells were in the adherent state (Additional file 1: Fig. S5). To further confirm the position of the NPs, posAuNPs were combined with RITC-DOPA-PEI, which confirmed that the treated NPs had adhered to the cell surface. In addition, we confirmed the attachment of NPs to the surface of C28/I2 cells treated with posAuNP@UCNPs at a ratio of posAuNP to UCNP of 1:5 using CellSEM. TEM and SEM confirmed that all NPs were bound to the cell membrane.

Next, we performed time-lapse live imaging to confirm whether UCPPin occurred when cells were treated with posAuNP@UCNPs (ratio 1:5). After attaching C28/I2 cells to the dish for confocal laser scanning microscopy (CLSM), the plasma membrane (PM) was dyed, and the sample was irradiated at 980 nm with a long-distance irradiator (Additional file 1: Fig. S6). Images were taken for up to 180 cycles every 6.5 s (Fig. 4B). When UCNPs are irradiated with 980 nm of light, the light energy is transferred to the AuNPs in solution. The thermal energy generated by this process results in vapor nanobubbles (VNBs), which make small holes in biological membranes as they pass through the membranes. The transfer of substances by transient perforation into the PM using light is called photoporation. In this study, we used a modified method employing UCNP-coated AuNPs termed upconverting-photon quenching-mediated perforation influx (UCPPin). At 0 s, posAuNP@UCNPs were bound to the cell surface; and at 139 s, we observed a decrease in the integrity of the PM and the formation of pores in the area indicated by the white arrow. The pores were stable for about 20 s, and the integrity of PM was restored at 159 s. The voxel view image on the right is a three-dimensional representation of images captured in real-time and accumulated over time. It was observed that posAuNP@UCNPs (red) were attached to PM (green) and pores were formed (white-filled arrows), and then PM was re-sealed (white hollow arrows) again. At 152 s, line profiling of the PM in the white arrow portion was performed, and the diameter of the generated pore was measured and is shown in the graph in Fig. 4C. These results confirmed that posAuNP@UCNPs attach to the cell membrane surface and form pores on the membrane surface under NIR irradiation.

To further confirm pore formation and plasma membrane re-sealing, we measured the intracellular calcium concentration in C28/I2 cells (Fig. 4D). According to the literature, plasma membrane damage induces calcium influx, which increases intracellular calcium levels and initiates plasma membrane re-sealing. Intracellular calcium has been shown to increase lysosomal exocytosis, resulting in membrane re-sealing [56–58]. Interpreting the results accordingly, it can be inferred that pore formation does not occur in the CONT group as calcium influx is not induced. On the other hand, in the posAuNP@UCNPs group, a rapid calcium influx was observed from 400 s, indicating that it was due to pore formation. Additionally, it can be inferred that calcium influx decreases starting at 800 s, indicating that calcium-dependent plasma membrane re-sealing is occurring. The calcium-free medium showed that both groups remained at low calcium concentrations (Additional file 1: Fig. S7). Based on these results, we conclude that treatment of C28/I2 cells with posAuNP@UCNPs 1:5 results in transient cell perforation followed by calcium-dependent PM re-sealing.

Several screenings were performed to determine the conditions for optimal UCPPin efficiency. First, we used staining with the impermeable fluorescent dyes propidium iodide (PI) and calcein to determine the optimum ratio of AuNPs to DOPA-PEI (DP) for posAuNPs formation (Additional file 1: Fig. S8A). The ratios of AuNP to DP tested were 1:0.5, 1:0.7, and 1:0.9, respectively. The efficiencies of posAuNPs without UCNP (1:0) and with UCNP (1:1) were compared. The results showed that the higher the DP ratio, the higher the number of PI-stained cells, indicating an increase in the number of dead cells. Calcein staining, on the other hand, was hardly detected as an indicator of living cells. This means that cells that have been stained with PI but have not been stained with calcein need weaker stimulation because it means that hyper-perforation occurs due to strong stimulation, resulting in death without being re-sealed of PM. UCPPin efficiency with and without UCNP at the same DP ratio was compared. Calcein-positive cells were observed in the UCNP (1:1) group, while the number of PI-positive cells was slightly lower in this group than in the (1:0) group. The DP ratio of 0.5 was considered too small since almost no PI-stained cells were detected, and the DP ratio of 0.9 resulted in too many dead cells. Therefore, in this study, the DP ratio was determined to be 0.7.

Second, a screening step was performed to determine the optimal NP incubation time before LED irradiation (Additional file 1: Fig. S8B). A comparison of the results obtained using a UNCP ratio of 1 between 0 to 20 min revealed that the number of dead cells increased with increasing incubation time. Thus, the optimal incubation time was determined to be up to 10 min.

Third, a screening step was performed to determine the optimal dose of posAuNP (Additional file 1: Fig. S9). We confirmed that as the amount of posAuNP increased, the number of dead cells also increased, and posAuNP 11.6 µg resulted 48% dead cells. However, since the number of dead cells decreased slightly when UCNP was combined with posAuNP in the previous experiment, we were decided not to keep the posAuNP dose too low and determined the optimal dose as10 µg.

**Fig. 5:**
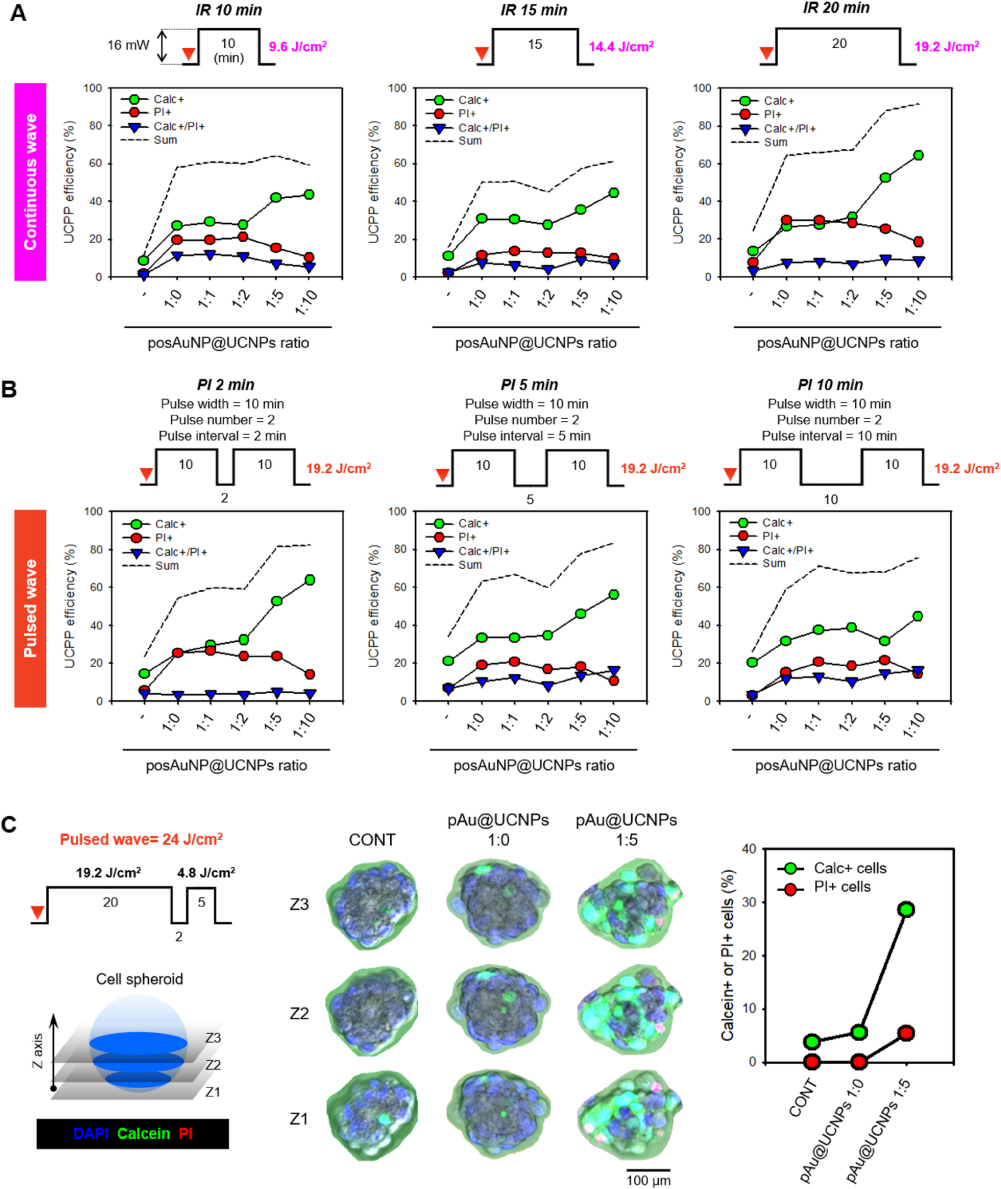
Comparison of UCPPin efficiency of posAuNP@UCNPs under different IR conditions in C28/I2 cell suspensions and spheroids. **A** UCPPin efficiency under continuous and pulsed IR analyzed in suspended cells. The percentage of Calc + cells indicate successful UCPPin, PI + cells indicate dead cells, and Calc + /PI + cells indicates that UCPPin occurred but the cells were damaged. **B** UCPPin efficiency under pulsed IR at different pulse intervals (PI) analyzed in suspended cells. The pulse width (PW) and pulse number (PN) were fixed at 10 min and 2, respectively. **C** Comparison of UCPPin efficiency between posAuNP@UCNPs 1:0 and 1:5 analyzed in spheroids under pulsed IR. Scale bar, 100 μm

An analysis was performed to determine the optimal UCNP ratio for UCPPin using posAuNP@UCNPs. First, the efficiency of UCPPin was confirmed by irradiating continuous waves at 980 nm of 0 min, 10 min, 15 min, and 20 min at an intensity of about 16 mW (Fig. 5A and Additional file 1: Fig. S10). As the irradiation time increased, the numbers of calcein-positive cells and PI-positive cells increased. However, as the UCNP ratio increased, the number of PI-positive cells decreased. Higher UCPPin efficiencies were observed at posAuNP to UCNP ratios of 1:1, 1:2, 1:5, and 1:10 than at 1:0. The efficiency was particularly high at ratios of 1:5 or greater with an irradiation time of 20 min (19.2J/cm^2^). A high efficiency of approximately 63% was confirmed under these conditions. The viabilities at each ratio and stage are shown in Additional file 1: Fig. S11.

Next, we examined the optimal pulse interval (PI) (Fig. 5B). The pulse width (PW) and pulse number (PN) were fixed at 10 min and 2, respectively. The PI was set at 2, 5, and 10 min for comparison. The 2 min PI group showed high UCPPin efficiency similar to that of a continuous irradiation time of 20 min, but the UCPPin efficiency decreased as the PI increased. At AuNP to UCNP ratios of 1:5 and 1:10, the UCPPin efficiency was high with no significant difference in efficiency between these two ratios; however, since processing a small amount is more appropriate than that of a large amount, the 1:5 ratio was used. As the irradiation time increased, the electromagnetic energy applied to cells accumulated, resulting in increased membrane disruption, dead cells, and higher UCPPin efficiency. However, the longer the PI, the weaker the accumulation of energy by cells; therefore, the number of dead cells and UCPPin efficiency showed a decreasing pattern.

The longer the irradiation time, the greater the accumulation of electromagnetic energy applied to the cells, resulting in increased membrane disruption, dead cell, and UCPPin efficiency. Conversely, the longer the PI, the weaker the accumulation of energy applied to the cells, resulting in a decrease in the number of dead cells and UCPPin.

Finally, an experiment was conducted to verify whether the pulsed wave could be applied to cell spheroids (Fig. 5C). A cell spheroid with a diameter of 150 μm was produced and pulsed at 24 J/cm2 (PW = 20 min, PN = 2, PI = 2 min, PW = 5 min). Images were captured using CLSM. The CONT and 1:0 groups showed an UCPPin efficiency of less than 5%. However, the 1:5 group showed an efficiency of about 30%, indicating high UCPPin efficiency. Similar results were confirmed using larger spheroids (Additional file 1: Fig. S12).

**Fig. 6:**
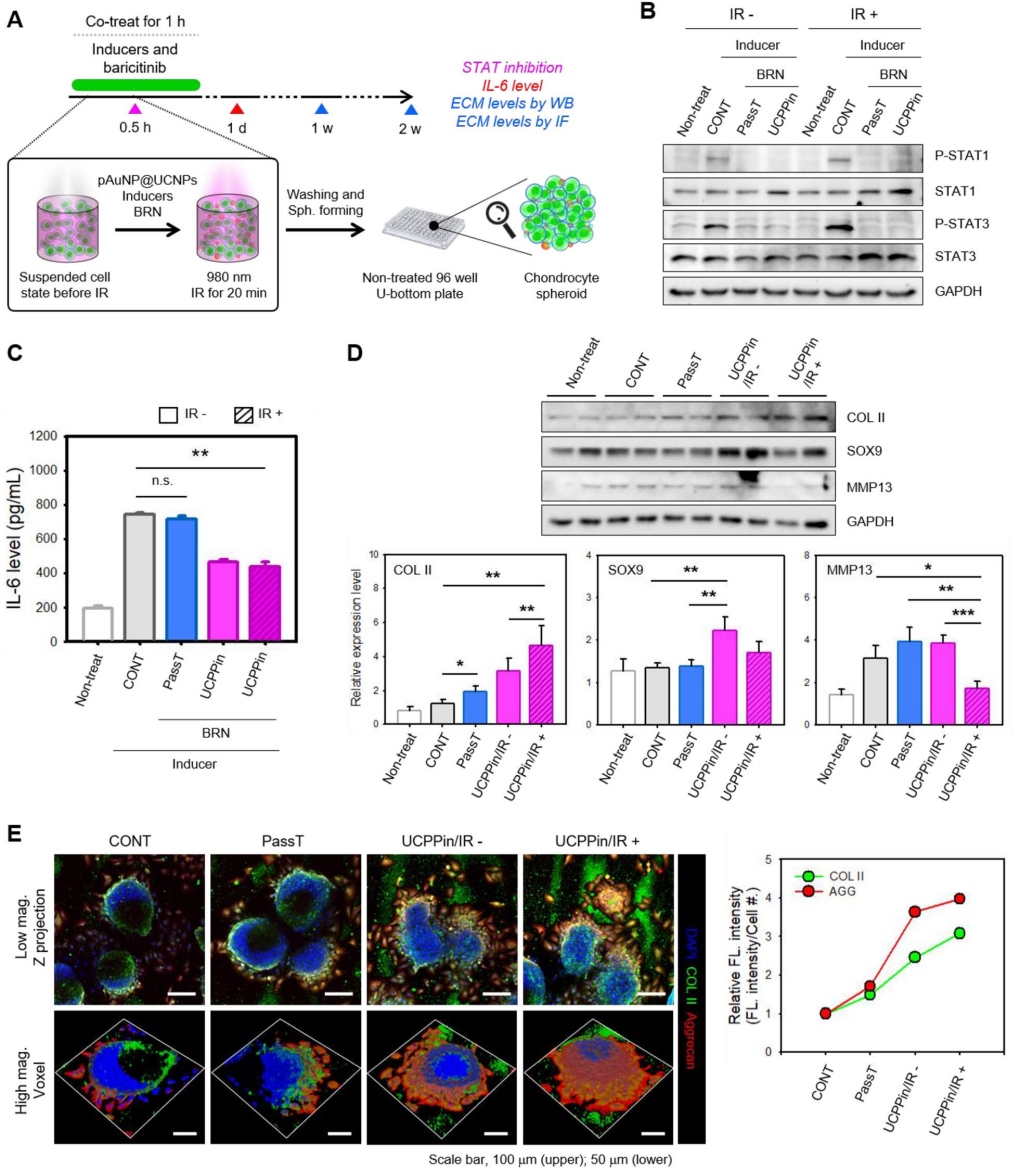
Comparison of therapeutic efficacy between UCPPin and PassT for intracellular delivery of baricitinib in an in vitro model of osteoarthritis. **A** Schematic diagram of the experimental procedure. **B** Inhibition of STAT1 and STAT3 phosphorylation by baricitinib analyzed by western blotting. **C** Evaluation of IL-6 levels using different intracellular delivery methods on Day 1. **D** Extracellular matrix (ECM) expression levels analyzed by western blotting **E** and immunofluorescence at Week 1 and Week 2, using different intracellular delivery methods

It is generally known that as osteoarthritis progresses, the expression level of proinflammatory cytokines such as IL-6 increases. Additionally, IL-6 signaling has been reported to be actively involved in osteoarthritis pathology and to be a factor in cartilage degradation [59–61]. Therefore, in this study, baricitinib (BRN), an inhibitor of STAT1 and 3 phosphorylation, was used as a therapeutic agent to specifically target STAT-IL-6 signaling. An in vitro 3D model was treated with posAuNP@UCNPs (1:5) for the delivering foreign substances into cells by posAuNP@UCNPs, and to test for passive transport (PassT), another method for delivering foreign substances through cell membranes. Suspended C28/I2 cells were treated with posAuNP@UCNPs, OA inducers (LPS and IL-6), and BRN. The solution was incubated at 4 °C for 10 min, irradiated with 980 nm LED for 20 min, 3D cultured, and analyzed for STAT inhibition, IL-6 concentration, and ECM expression levels (Fig. 6A). The level of inhibition of STAT phosphorylation by BRN was confirmed by WB (Fig. 6B) and used to determine whether BRN was delivered into cells through UCPPin and PassT. BRN was effectively delivered by both UCPPin and PassT into cells with or without IR, as shown by the inhibition of STAT phosphorylation. Viability was measured on Day 1 (Additional file 1: Fig. S12) and no difference in viability was observed between groups. However, the expression of IL-6, an inflammatory cytokine acting downstream of STAT, was different between cells delivered with BRN by UCPPin and those delivered with BRN by PassT. IL-6 expression of PassT-BRN delivered cells was similar to that of control cells (CONT), whereas it was lower in UCPPin-BRN delivered cells (by about 40–45%) (Fig. 6C). Thus, UCPPin would appear to deliver BRN into cells more efficiently than PassT, as evidenced by the level of inhibition of IL-6 expression.

ECM expression levels were analyzed by western blot and immunofluorescence staining at 1 and 2 weeks, respectively. At week 1, the expression levels of COLII, SOX9, and MMP13 were analyzed using duplication for each experimental group. SOX9 is a major transcription factor in the cartilage formation process, COLII is a representative ECM, and MMP13 is an enzyme that decomposes ECM such as COL II, so it was necessary to check the protein expression of these markers. COL II expression was significantly higher in the UCPPin group than in the CONT and PassT groups, and especially in the UCPPin/IR + group (Fig. 6D). MMP13 expression was similar between CONT, PassT, and UCPPin/IR cells, but it was reduced in UCPPin/IR + cells. SOX9 showed the highest expression in the UCPPin/IR- group, but since it functions to initiate differentiation with a transcription factor that acts at all stages of the cartilage formation process, a measure of the possibility of differentiation is more suitable than a measure of the degree of differentiation. However, UCPPin/IR + group is also meaningful because it is expressed higher than other groups like IR-group.

Figure 6E shows that COL II and Aggrecan (AGG) expression increased in the UCPPin group in the second 2nd week. COL II is expressed from the center to the inside of the spheroid in a 3D environment, and AGG is expressed on the outside of the spheroid. Taken together, these results demonstrate that UCPPin/IR-mediated delivery of BRN is more effective in promoting ECM expression and tissue formation than PassT-mediated delivery of BRN, and that IR further promotes these effects of UCPPin/IR-mediated delivery of BRN.

## Conclusion

In this study, the morphological and physical properties of posAuNP@UCNPs were characterized and the changes that appeared by irradiating 980 nm light on posAuNP@UCNPs were evaluated. Transient perforation and re-sealing of the PM was observed after treatment with posAuNP@UCNPs. Furthermore, this study compared the efficiency of posAuNP@UCNPs for UCPPin according to IR time and type, and analyzed their effects on C28/I2 cell suspensions and spheroids. Finally, we compared the therapeutic efficacy of UCPPin and PassT for intracellular delivery of baricitinib in an in vitro 3D model of OA. We showed that UCPPin by posAuNP@UCNP nanocomposites was better for substance delivery than PassT. Although it delivered BRN, a small molecule, this study suggests that UCPPin is not only effective for substance delivery, but is also applicable for the treatment of OA. In the future, UCPPin could be used for the delivery of RNAs and other macromolecules, which would further expand its scope for use in clinical treatments.

### Supplementary Information

**Additional file 1: Figure S1. **FTIR spectral of PEI and DOPA-PEI. **Figure S2. **TEM images and FTIR spectra of posAuNP compared with AuNP. **Figure S3. **Extinction and emission profiling of posAuNPs and UCNP, respectively. Figure S4. Characterization of the 980 nm LED irradiator **A** and absorbance curves of LED-irradiated posAuNP@UCNPs nanocomposites (**B**). **Figure S5.** Confocal **A** and CellSEM **B** images of posAuNP@UCNPs-treated C28/I2 cells. **Figure S6. **Long-distance irradiating system of LED for time-lapse imaging. **Figure S7. **Time-lapse imaging for estimating the intracellular calcium level during PM re-sealing. Figure S8. Determination of the AuNP:DOPA-PEI ratio and incubation time analyzed by UCPPin efficiency. **Figure S9. **Determination of posAuNP dose analyzed by UCPPin efficiency. **Figure S10. **Evaluation of UCPPin efficiency according to IR time and pulse type. **Figure S11.** Viability test during the UCPPin process at each step. **Figure S12.** Evaluation of UCPPin efficiency in C28/I2 spheroids. **Figure S13.** Viability of C28/I2 cells treated with UCPPin or PassT for 24 hours in OA 3D models.

## Data Availability

No data was used for the research described in the article.
