# Supplementary material for: Upconverting-photon quenching-mediated perforation influx as an intracellular delivery method using posAuNP@UCNPs nanocomposites for osteoarthritis treatment
Source: Nano Converg. 2024 Jan 3;11:1. doi: 10.1186/s40580-023-00409-y (PMC10764707; doi:10.1186/s40580-023-00409-y)
Supplement: Supplementary file 1 — Additional file 1: Figure S1. FTIR spectral of PEI and DOPA-PEI. Figure S2. TEM images and FTIR spectra of posAuNP compared with AuNP. Figure S3. Extinction and emission profiling of posAuNPs and UCNP, respectively. Figure S4. Characterization of the 980 nm LED irradiator A and absorbance curves of LED-irradiated posAuNP@UCNPs nanocomposites (B). Figure S5. Confocal A and CellSEM B images of posAuNP@UCNPs-treated C28/I2 cells. Figure S6. Long-distance irradiating system of LED for time-lapse imaging. Figure S7. Time-lapse imaging for estimating the intracellular calcium level during PM re-sealing. Figure S8. Determination of the AuNP:DOPA-PEI ratio and incubation time analyzed by UCPPin efficiency. Figure S9. Determination of posAuNP dose analyzed by UCPPin efficiency. Figure S10. Evaluation of UCPPin efficiency according to IR time and pulse type. Figure S11. Viability test during the UCPPin process at each step. Figure S12. Evaluation of UCPPin efficiency in C28/I2 spheroids. Figure S13. Viability of C28/I2 cells treated with UCPPin or PassT for 24 hours in OA 3D models. [file 40580_2023_409_MOESM1_ESM.docx]

**Upconverting-photon Quenching-mediated Perforation Influx as an Intracellular Delivery Method using posAuNP@UCNPs Nanocomposites for Osteoarthritis treatment**

Hye Jin Kim, Hui Bang Cho, Hye-Ryoung Kim, Sujeong Lee, Ji-in Park, Keun-Hong Park*

Laboratory of Nano-regenerative Medicine, Department of Biomedical Science, College of Life Science, CHA University, CHA Biocomplex, Sampyeong-Dong, Bundang-gu, Seongnam-si, 13488, Republic of Korea

**Additional file 1: Figures**

**
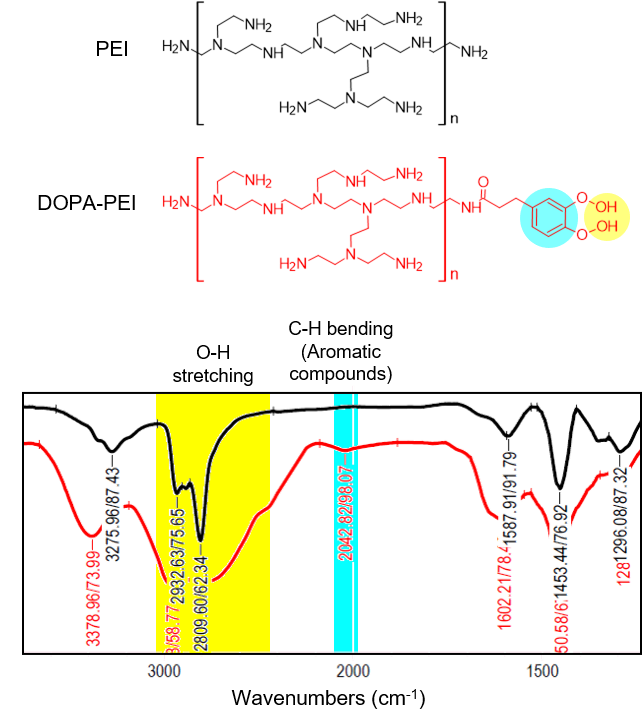
**

**Figure S1. FTIR spectral of PEI and DOPA-PEI.**

**
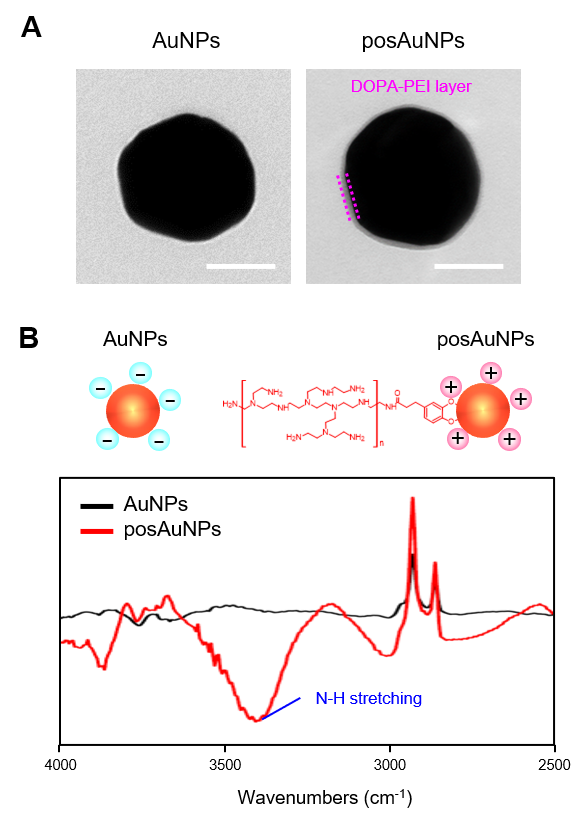
**

**Figure S2. TEM images and FTIR spectra of posAuNP compared with AuNP.**

**
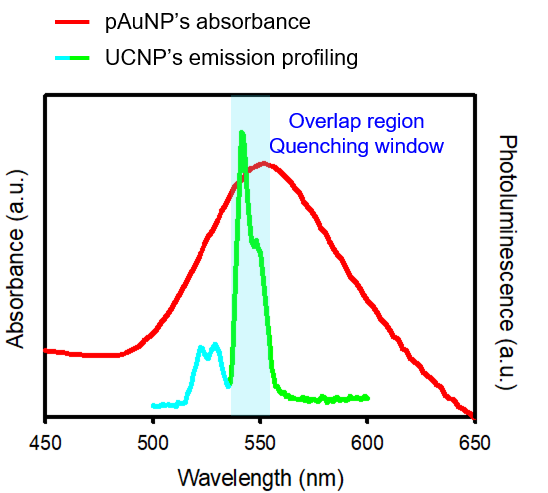
**

**Figure S3. Extinction and emission profiling of posAuNPs and UCNP, respectively.**

**
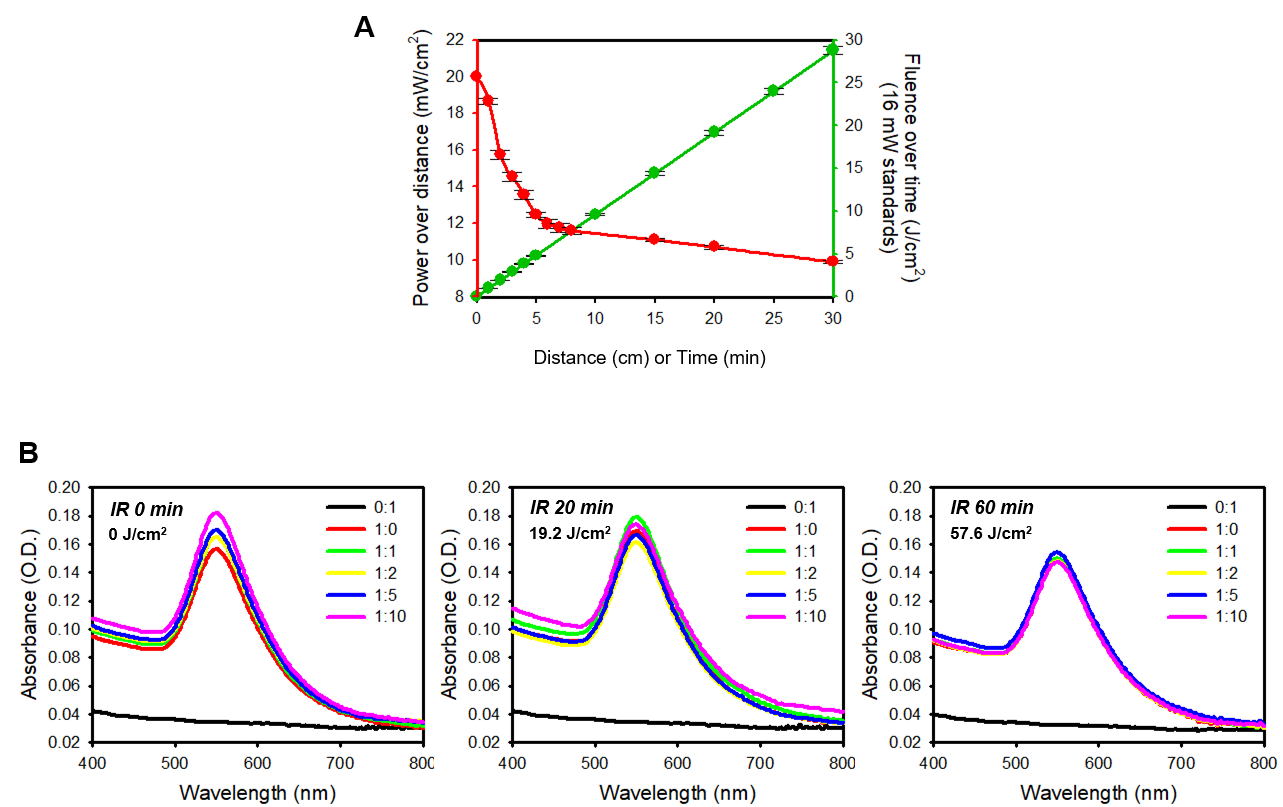
**

**Figure S4. Characterization of the 980 nm LED irradiator (A) and absorbance curves of LED-irradiated posAuNP@UCNPs nanocomposites (B).**

**
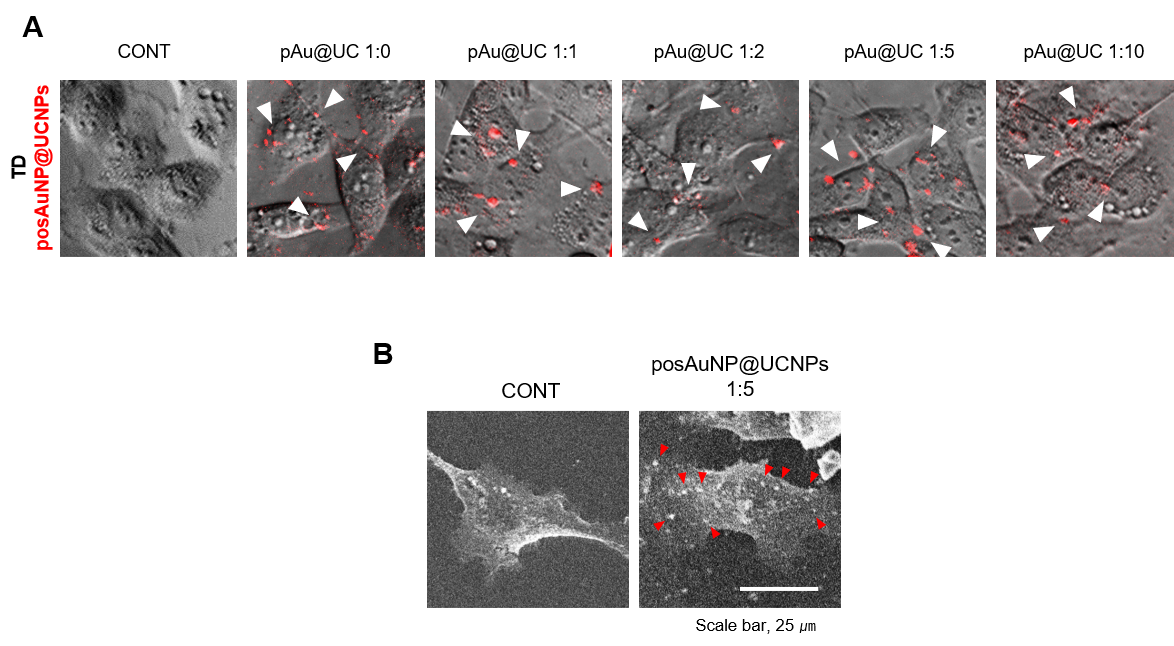
**

**Figure S5. Confocal (A) and CellSEM (B) images of posAuNP@UCNPs-treated C28/I2 cells.**

**
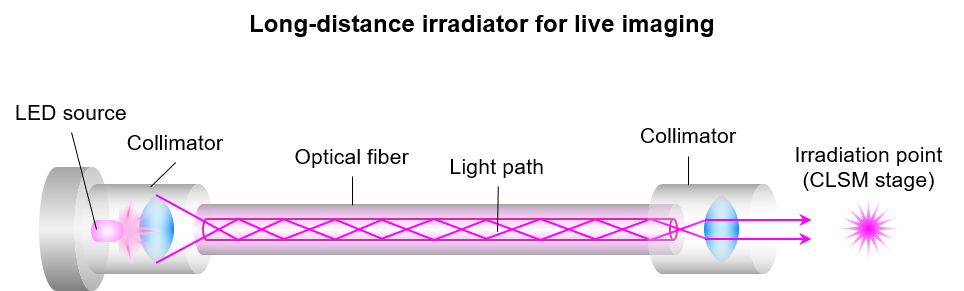
**

**Figure S6. Long-distance irradiating system of LED for time-lapse imaging.**

**
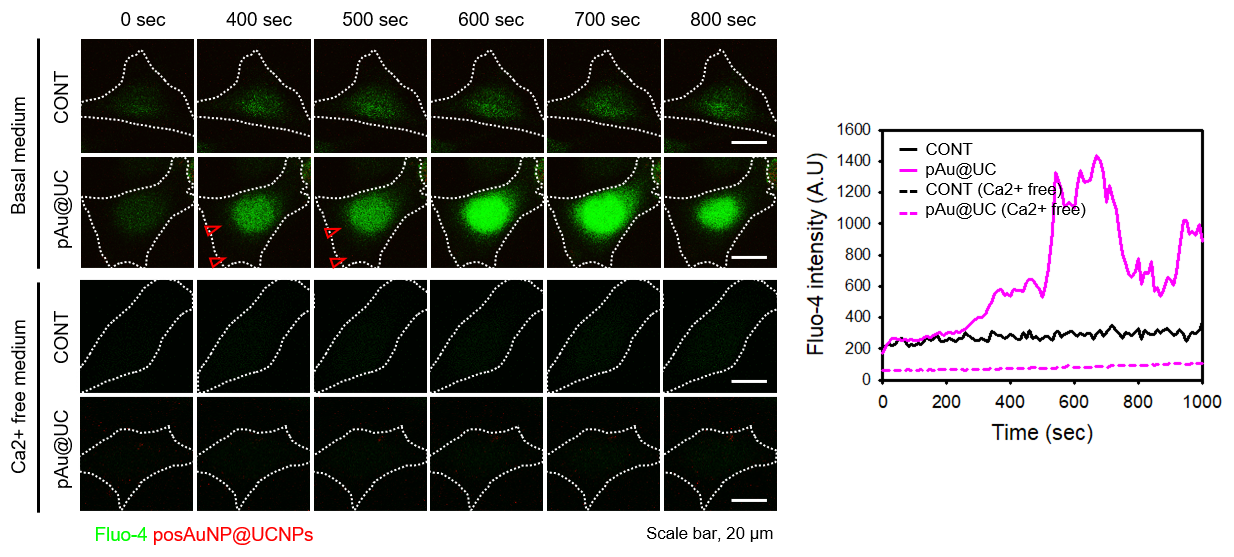
**

**Figure S7. Time-lapse imaging for estimating the intracellular calcium level during PM re-sealing.**

**
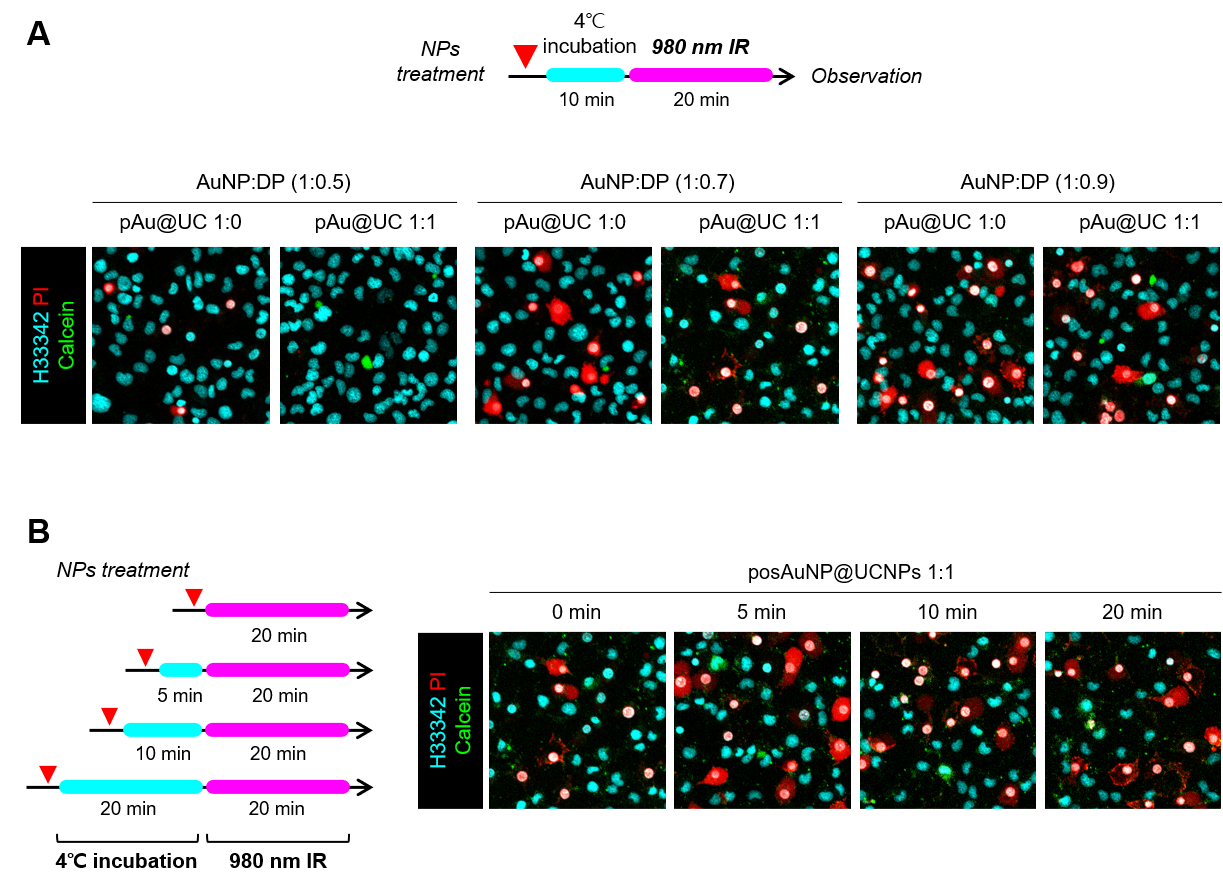
**

**Figure S8. Determination of the AuNP:DOPA-PEI ratio and incubation time analyzed by UCPPin efficiency.**

**
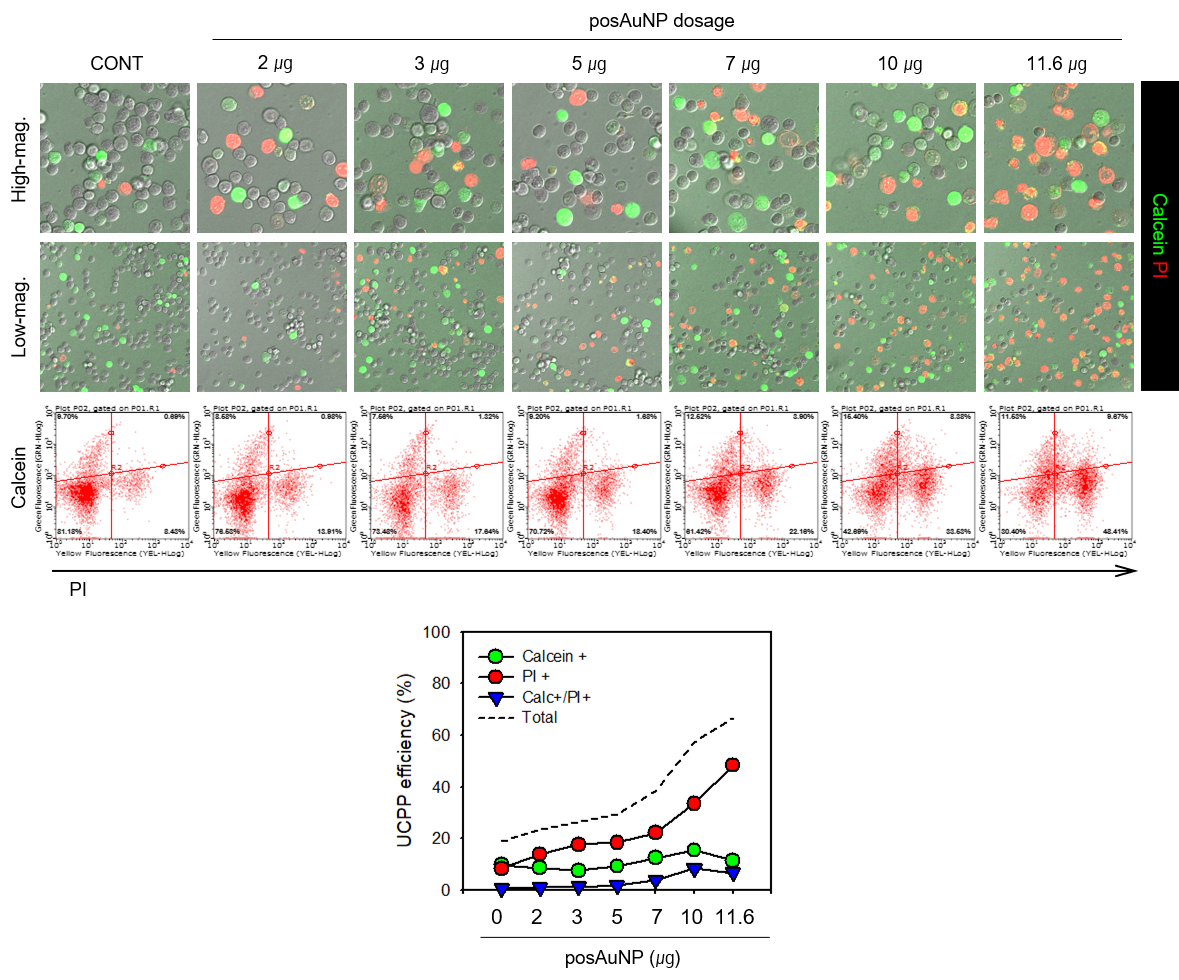
**

**Figure S9. Determination of posAuNP dose analyzed by UCPPin efficiency.**

**
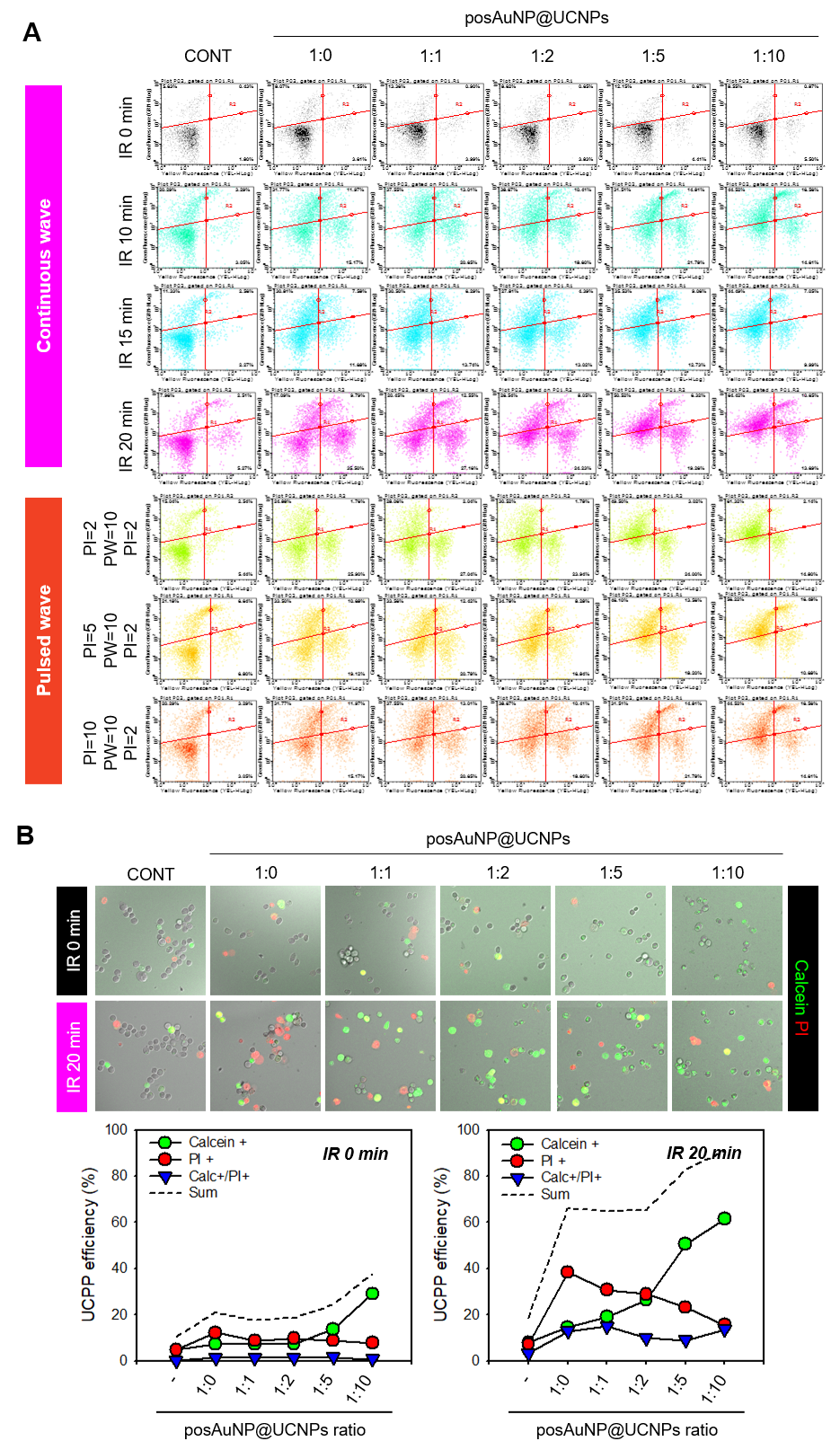
**

**Figure S10. Evaluation of UCPPin efficiency according to IR time and pulse type.**

**
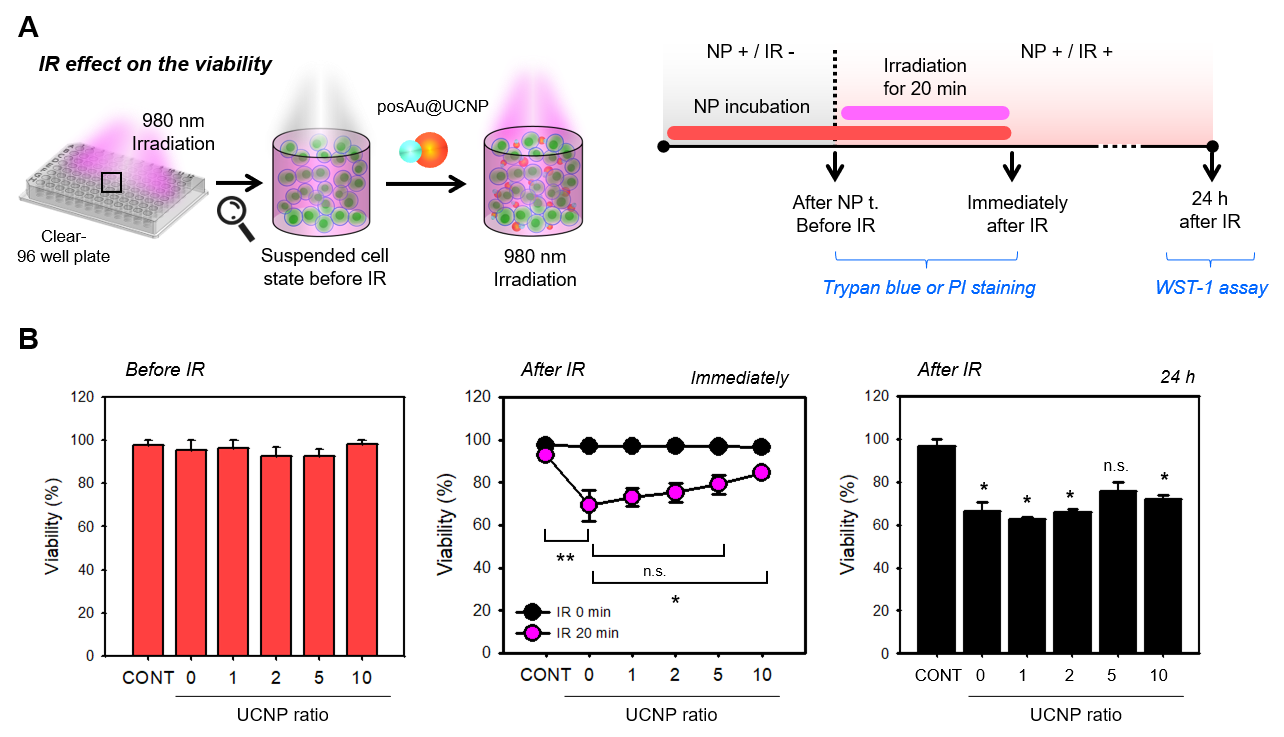
**

**Figure S11. Viability test during the UCPPin process at each step.**

(A) Schematic diagram of the experimental procedure. (B) Cell viabilities.


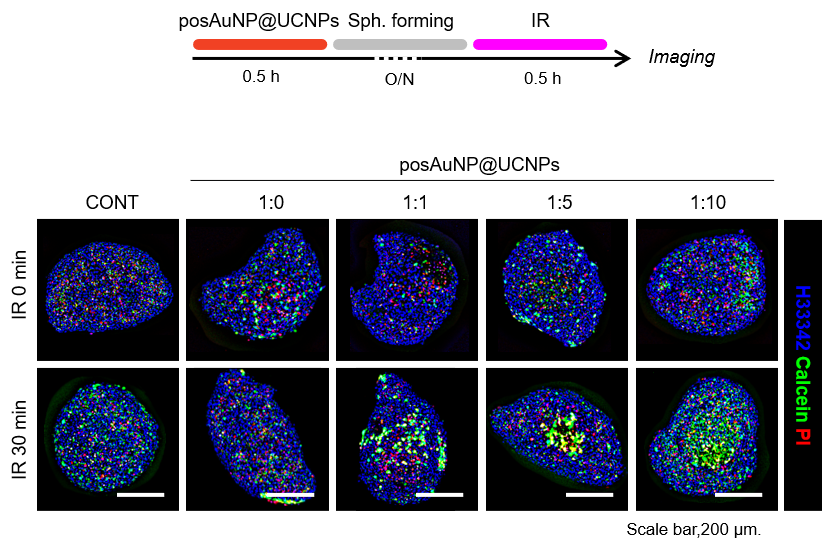


**Figure S12. Evaluation of UCPPin efficiency in C28/I2 spheroids.**

**
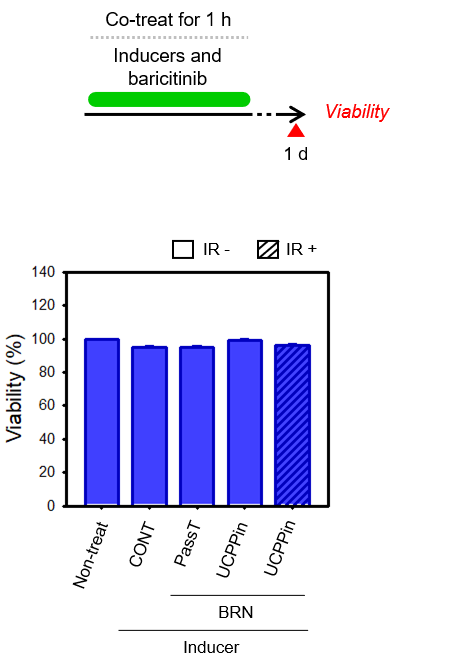
**

**Figure S13. Viability of C28/I2 cells treated with UCPPin or PassT for 24 hours in OA 3D models.**
